# Radical Intermediates in Photoinduced Reactions on TiO_2_ (An EPR Spin Trapping Study)

**DOI:** 10.3390/molecules191117279

**Published:** 2014-10-28

**Authors:** Dana Dvoranová, Zuzana Barbieriková, Vlasta Brezová

**Affiliations:** Institute of Physical Chemistry and Chemical Physics, Faculty of Chemical and Food Technology, Slovak University of Technology in Bratislava, Radlinského 9, Bratislava SK-812 37, Slovakia

**Keywords:** titanium dioxide, hydroxyl radical, reactive oxygen species, EPR spectroscopy, spin trapping technique, sterically hindered amines

## Abstract

The radical intermediates formed upon UVA irradiation of titanium dioxide suspensions in aqueous and non-aqueous environments were investigated applying the EPR spin trapping technique. The results showed that the generation of reactive species and their consecutive reactions are influenced by the solvent properties (e.g., polarity, solubility of molecular oxygen, rate constant for the reaction of hydroxyl radicals with the solvent). The formation of hydroxyl radicals, evidenced as the corresponding spin-adducts, dominated in the irradiated TiO_2_ aqueous suspensions. The addition of ^17^O-enriched water caused changes in the EPR spectra reflecting the interaction of an unpaired electron with the ^17^O nucleus. The photoexcitation of TiO_2_ in non-aqueous solvents (dimethylsulfoxide, acetonitrile, methanol and ethanol) in the presence of 5,5-dimethyl-1-pyrroline *N*-oxide spin trap displayed a stabilization of the superoxide radical anions generated via electron transfer reaction to molecular oxygen, and various oxygen- and carbon-centered radicals from the solvents were generated. The character and origin of the carbon-centered spin-adducts was confirmed using nitroso spin trapping agents.

## 1. Introduction

Among the materials previously studied as potential photocatalysts, titanium dioxide meets the criteria for industrial-scale utilization. Stability, low cost, relatively low toxicity and appropriate photocatalytic activity predispose TiO_2_ to a wide range of applications in various areas (gas sensors, photocatalysts, solar cells, thin film capacitors, self-cleaning surfaces, *etc.*) [1,2,3,4,5,6]. Especially attractive are nowadays the prospects of titania photocatalysts applications in the remediation of polluted water, soil and air, or in unconventional organic syntheses [7,8,9,10,11,12,13]. Consequently, all titanium dioxide polymorphs (anatase, brookite, rutile) have been intensively studied regarding their ability to produce, upon UVA photoexcitation, electron (e^−^) and hole (h^+^) pairs further involved in the consecutive chemical reactions [1,2,5,14,15,16]. In general, the photoactivity of TiO_2_ is determined by the processes of electron/hole pair generation, recombination, interfacial transfer and by the surface reactions of these charge carriers with the species adsorbed on the surface of the photocatalyst [1,2,5,10,15,16,17]. The photoinduced processes on TiO_2_ nanoparticles upon ultra-band gap irradiation are also well influenced by the bulk structure, surface properties and the electronic structure of the photocatalyst [5]. The reactions of photogenerated holes with the adsorbed hydroxide anions and water molecules lead to the formation of highly reactive hydroxyl radicals, which, together with the hole itself, can initiate the oxidative degradation of organic pollutants down to water and carbon dioxide [1,2,5,10,16]. The efficient production of hydroxyl radicals and their non-selective reactions with organic and inorganic pollutants represent a crucial point considering the application of photocatalytic processes in water and air purification [1]. Recent investigations have revealed different mechanisms on anatase and rutile surfaces [18], as well as the role of surface-bridging oxygens of TiO_2_ on the ^•^OH formation associated with the oxidation of surface hydroxide anions and water molecules by the photogenerated holes [19,20]. The presence of molecular oxygen also plays a substantial role in the photoinduced processes on irradiated TiO_2_ surfaces, as it enables an effective charge carriers separation. The electrons trapped transiently on the surface or on the next-to-surface defects can react with the adsorbed oxygen molecules [21,22,23]. The consecutive reactions of the so generated O_2_^•−^ are influenced by the solvent properties [24]. Although the superoxide radical anion is quite stable in the aprotic solvents [25], in aqueous solutions the reaction with protons is favorable, and hydrogen peroxide is formed and involved in further photocatalytic processes, Equations (1)–(6) [26]:

O_2_^•−^ + H^+^ → ^•^O_2_H (1)

2 ^•^O_2_H → H_2_O_2_ + O_2_ (2)

H_2_O_2_ + O_2_^•−^ → ^•^OH + O_2_ + OH^−^ (3)

(4) H2O2→hv2OH•

H_2_O_2_ + e^−^ → ^•^OH + OH^−^ (5)

H_2_O_2_ + h^+^ + OH^−^ → H_2_O + ^•^O_2_H (6)

Consequently, superoxide radical anion and hydrogen peroxide as the most important products of the molecular oxygen reduction play an important role in the complex mechanism of Reactive Oxygen Species (ROS, e.g., ^•^OH, ^•^O_2_H or singlet oxygen) generation on the irradiated TiO_2_ surfaces [1,18,26,27,28].

**Figure 1:**
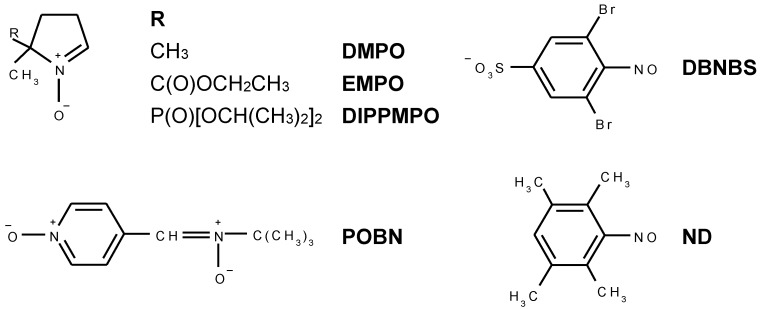
Overview of the spin trapping agents applied in *in situ* EPR investigations.

EPR spectroscopy occupies an exclusive position in the investigation of titania photocatalysts, providing a characterization of paramagnetic centers produced via the trapped photogenerated electrons and holes [29,30,31,32,33,34,35,36], and of titanium dioxide materials with transition-metal ions doping [17,34]. A majority of the research exploiting EPR spectroscopy deals with the investigation of reactive radical intermediates produced in the irradiated TiO_2_ particulate systems where the application of an indirect spin trapping technique is inevitable [37,38,39,40,41,42,43,44,45]. This method is based on the chemical reaction of a diamagnetic spin trap (ST) with a short-lived radical, producing a more stable nitroxide radical, *i.e.*, spin-adduct, using nitrones, *N*-oxides and nitroso compounds as the spin trapping agents (Figure 1). Spin traps possessing *N*-oxide and nitrone groups are mainly applied in the identification of hydroxyl radicals generation, as well as other oxygen-, nitrogen- and sulfur-centered reactive radicals, however the information on the structure of carbon-centered radicals trapped with these agents is limited, and the application of nitroso spin traps is necessary to bring the knowledge on other nuclei in the vicinity of the trapped carbon [46,47]. Successful assignment of measured EPR spectra of spin-adducts requires a thorough interpretation of the acquired data and careful choice of the spin trapping agent for the specific experimental conditions [48].

In the literature hydroxyl radicals are frequently declared as the most important reactive species generated upon TiO_2_ irradiation, in spite of the addition of organic co-solvent to the reaction systems, which effects the character and amount of radicals formed. The main aim of our study was to point on the formation of radical intermediates in aqueous TiO_2_ suspensions, as well as in suspensions prepared in an organic solvent (DMSO, acetonitrile, methanol, ethanol) and thus provide a straightforward comparison of the radical species detected under UVA exposure of TiO_2_ suspended in water and in aprotic and protic polar solvents exploiting the *in situ* EPR spin trapping technique. The oxidation of sterically hindered amines to the corresponding nitroxide radicals via ROS photogenerated in the aqueous and acetonitrile TiO_2_ suspensions was also monitored by *in situ* EPR spectroscopy.

## 2. Results and Discussion

### 2.1. Spin Trapping in the Aqueous TiO_2_ Suspensions

**Figure 2:**
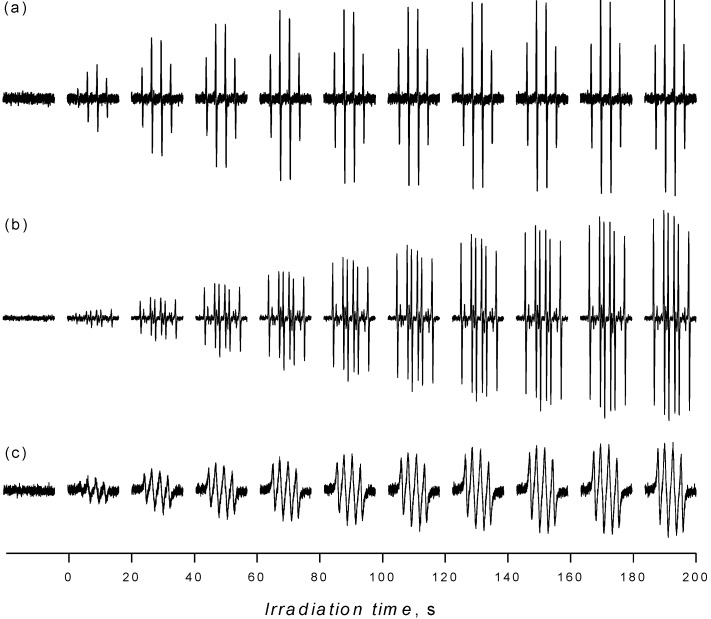
The sets of individual EPR spectra (magnetic field sweep width, *SW* = 8 mT) monitored upon continuous UVA irradiation (λ_max_ = 365 nm; irradiance 15 mW∙cm^−2^) of aerated TiO_2_ P25 suspensions in the presence of spin trapping agent DMPO: (**a**) water; (**b**) mixed solvent water/dimethylsulfoxide (5:1 v:v); (**c**) acetonitrile. TiO_2_ concentration 0.167 mg∙mL^−1^, *c*_0,DMPO_ = 0.035 M.

Despite the fact that the detection of hydroxyl radicals upon UVA irradiation of the aerated aqueous TiO_2_ suspensions in the presence of 5,5-dimethyl-1-pyrroline *N*-oxide (DMPO) spin trap represents a frequently applied EPR technique [38,39,40,41,42,43,44,45,49], in order to bring the complete information on the radical species generated in the irradiated TiO_2_ in different media we also report the results of EPR spin trapping experiments in aqueous TiO_2_ suspensions using different spin trapping agents. As expected, immediately after the irradiation started, a typical four-line EPR signal attributed to ^•^DMPO-OH spin-adduct with spin Hamiltonian parameters (*a*_N_ = 1.497 mT, *a*_H_ = 1.477 mT; *g* = 2.0057 [50]) was generated in the system TiO_2_/DMPO/H_2_O/air, as is shown in Figure 2a. The concentration profile of ^•^DMPO-OH during the *in situ* EPR spin trapping experiments is strongly influenced by TiO_2_ loading, UVA radiation dose, as well as by the initial oxygen and spin trap concentrations [38,44].

The primary source of the ^•^OH radicals in the irradiated aqueous TiO_2_ suspensions is the oxidation of OH^−^ and H_2_O by the photogenerated holes, however further reactions of the reactive oxygen species generated in the system leading to ^•^OH cannot be excluded (Equations (1)–(6)). The EPR spin trapping technique is assumed to detect the photogenerated hydroxyl radicals on the photocatalysts’ surfaces, based on the previous comparison with the quantification of ^•^OH via fluorescence detection using the hydroxylation of terephthalic acid, by which the bulk ^•^OH are detected [43].

**Figure 3:**
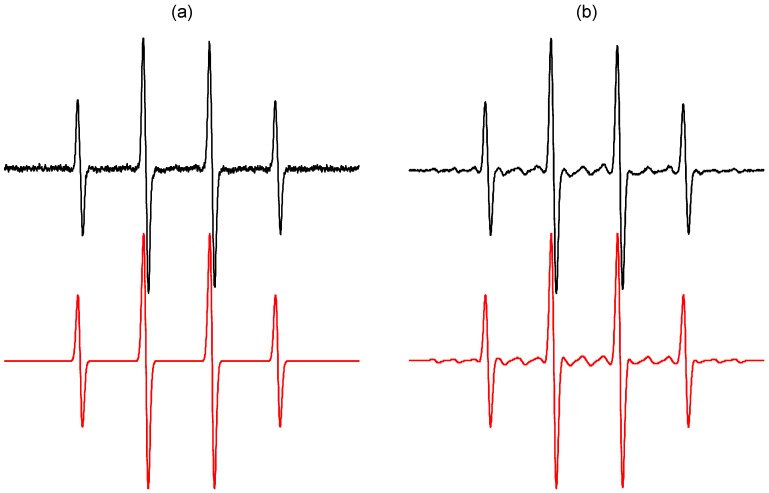
Experimental (**black**) and simulated (**red**) EPR spectra (*SW* = 8 mT) obtained upon irradiation (λ_max_ = 365 nm; irradiance 15 mW∙cm^−2^; exposure 400 s) of the aerated aqueous TiO_2_ P25 suspensions in the presence of spin trapping agent DMPO (TiO_2_ concentration 0.167 mg∙mL^−1^, *c*_0,DMPO_ = 0.035 M): (**a**) ordinary water; (**b**) water enriched with H_2_^17^O (13%–17% atom.). Simulation parameters (hfcc in mT): (a) ^•^DMPO–OH (*a*_N_ = 1.497, *a*_H_ = 1.477; *g* = 2.0057); (b) linear combination of spin-adducts, *i.e.*, ^•^DMPO–OH (relative concentration in %, 82) and ^•^DMPO-^17^OH (*a*_N_ = 1.494, *a*_H_ = 1.480, *a*_17O_ = 0.467; *g* = 2.0057; 18).

Figure 3 illustrates experimental and simulated EPR spectra of ^•^DMPO-OH measured in the TiO_2_ suspensions prepared either using ordinary water, or water enriched with the magnetically active ^17^O (13%–17% atom.) nucleus. The EPR spectrum depicted in Figure 3b is fully compatible with the presence of both spin-adducts, *i.e.*, ^•^DMPO-OH and ^•^DMPO-^17^OH [51], unambiguously identifying the adsorbed and close-to-surface water molecules as the source of hydroxyl radicals. Recently, we conducted EPR spin trapping experiments using aerated aqueous suspensions of Ti^17^O_2_ (containing up to 90% atom. ^17^O) with DMPO, and the EPR spectra of ^•^DMPO-OH corresponded to the interaction of one nitrogen nucleus (*a*_N_ = 1.492 mT) and one hydrogen nucleus (*a*_H_ = 1.476 mT) with an unpaired electron [52]. No evidence of a hyperfine coupling from ^17^O was found, consequently the lattice oxygens from TiO_2_ were excluded as the source of hydroxyl radicals trapped by DMPO under the given experimental conditions [52].

Previously, the possibility of a direct oxidation of the spin trapping agent DMPO via photogenerated holes to a radical cation DMPO^•+^, which subsequently reacts with water molecules forming a so-called imposter spin-adduct ^•^DMPO-OH, was supposed [41,43,44]. In addition, the degradation of the low-stability ^•^DMPO-O_2_H spin-adduct, theoretically also generated in the studied system, results in the ^•^DMPO-OH formation [53]. However, the generation of the surface hydroxyl radicals can be evidenced by the addition of dimethylsulfoxide (DMSO) to the aqueous TiO_2_ suspensions, since the rapid reaction of hydroxyl radicals with DMSO (Table 1) produces methyl radicals [54], detectable in the reaction with spin trap (ST) as the corresponding carbon-centered spin-adduct, Equations (7) and (8):

(CH_3_)_2_SO + ^•^OH → CH_3_(OH)SO + ^•^CH_3_ (7)

^•^CH_3_ + ST → ^•^ST-CH_3_ (8)

**Table 1:** Bimolecular rate constants for the reaction of hydroxyl radical with the selected solvents [54], decay constants and lifetime of singlet oxygen (^1^∆_g_) in selected solvents [55].

Solvent	$k_{{}^{•}\text{OH}}$, M^−1^∙s^−1^	$k_{{}^{1}O_{2}}$, s^−1^	*τ* (1/$k_{{}^{1}O_{2}}$), μs
Water	–	2.4 × 10^5^	4.2
Dimethylsulfoxide ^#^	7.0 × 10^9^	5.2 × 10^4^	19
Acetonitrile ^#^	2.2 × 10^7^	1.4 × 10^4^	71
Methanol ^#^	8.3 × 10^8^	1.1 × 10^5^	9
Ethanol ^#^	2.2 × 10^9^	7.9 × 10^4^	13

^#^: determined in aqueous solutions.

**Figure 4:**
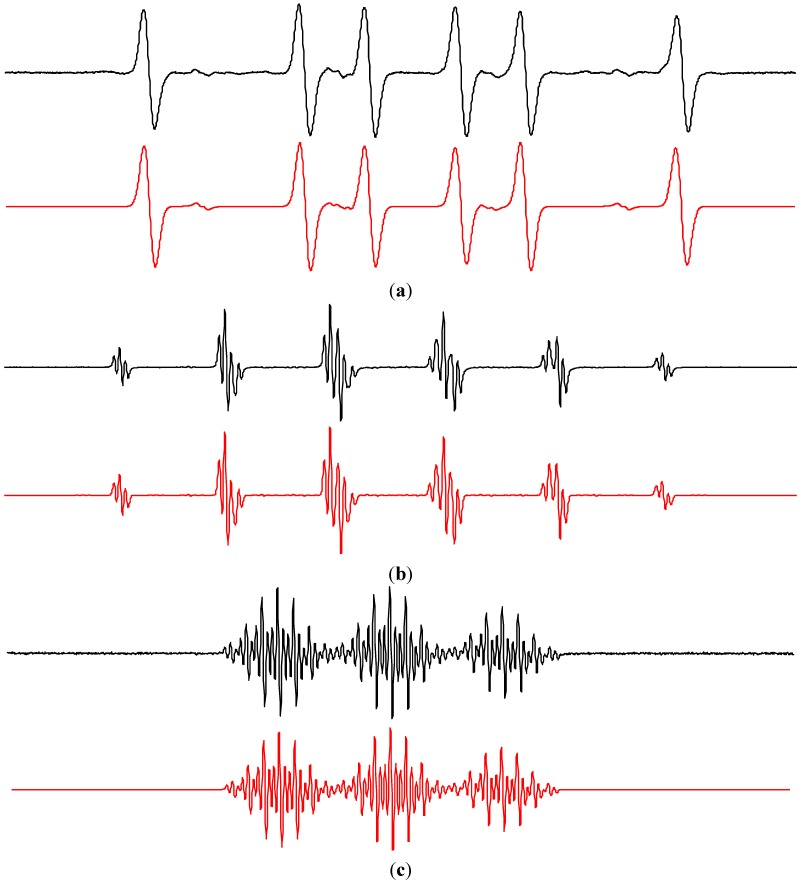
Experimental (**black**) and simulated (**red**) EPR spectra obtained upon irradiation (λ_max_ = 365 nm; irradiance 15 mW∙cm^−2^; exposure 400 s) of the aerated TiO_2_ P25 suspensions in mixed solvent water/DMSO (5:1 v:v) in the presence of various spin trapping agents (TiO_2_ concentration 0.167 mg∙mL^−1^): (**a**) DMPO (*SW* = 8 mT; *c*_0,DMPO_ = 0.035 M); (**b**) DBNBS (*SW* = 10 mT; *c*_0,DBNBS_ = 0.008 M); (**c**) DBNBS using DMSO-*d*_6_. The spin Hamiltonian parameters of corresponding spin-adducts are listed in Table 2. (a) ^•^DMPO-CH_3_ (relative concentration in %, 94), ^•^DMPO-OH (6); (b) ^•^DBNBS–CH_3_ (100); (c) ^•^DBNBS-CD_3_ (100).

Figure 2b shows the set of EPR spectra monitored during the exposure of TiO_2_/DMPO/air in mixed solvent water/DMSO (5:1 v:v). The EPR spectra obtained are more complex compared to those found in water suspensions (Figure 2a), and represent a superposition of the dominant six-line signal attributed to ^•^DMPO-CH_3_ and the low-intensity signal of ^•^DMPO-OH with slightly modified hyperfine coupling constants (hfcc) caused by the DMSO presence in the system [56]. The experimental and simulated EPR spectra found upon 400 s exposure are depicted in Figure 4a and the corresponding spin Hamiltonian parameters elucidated from the simulated spectra are summarized in Table 2.

Further experiments using 3,5-dibromo-4-nitrosobenzene sulfonate (DBNBS) spin trapping agent in aerated aqueous TiO_2_ suspensions containing DMSO or DMSO-*d*_6_ (water/DMSO, 5:1 v:v) unambiguously confirmed the generation of methyl radicals via the reaction of hydroxyl radicals with the solvent, as the EPR spectra monitored upon UVA irradiation are well-matched to ^•^DBNBS-CH_3_ or ^•^DBNBS-CD_3_ spin-adducts (Figure 4b,c), respectively, with the spin Hamiltonian parameters well correlated with literature data (Table 2).

**Table 2:** Spin Hamiltonian parameters (hyperfine coupling constants and *g*-values) of the spin-adducts elucidated from simulations of the experimental EPR spectra obtained upon UVA irradiation (λ_max_ = 365 nm) of aerated TiO_2_ P25 suspensions in water and water/dimethylsulfoxide mixed solvent (5:1 v:v) in the presence of the corresponding spin trapping agents.

Spin-Adduct	Hyperfine Coupling Constants(mT)		*g*-Value	Reference
	*a*_NO_	*a*_i_		
**Water**				
**^•^DMPO–OH**	1.497	*a*_H_^β^ = 1.477	2.0057	[50]
**^•^DMPO–^17^OH**	1.494	*a*_H_^β^ = 1.480; $a_{{}^{\mathrm{17}}O}$ = 0.469	2.0057	[51,57]
**^•^DMPO–N_3_**	1.481	*a*_H_^β^ = 1.426; *a*_N_ = 0.314	2.0057	[50]
***trans*-^•^EMPO–OH**	1.410	*a*_H_^β^ = 1.278; *a*_H_^γ^ = 0.066; *a*_H_^γ^ = 0.043	2.0056	[58,59]
***cis*-^•^EMPO–OH**	1.410	*a*_H_^β^ = 1.542	2.0056	[58,59]
**^•^EMPO_degr_**	1.514	*a*_H_^β^ = 2.187	2.0056	–
***trans*-^•^DIPPMPO–OH**	1.410	*a*_H_^β^ = 1.319; *a*_P_ = 4.692	2.0055	[59,60]
***cis*-^•^DIPPMPO–OH**	1.646	*a*_H_^β^ = 1.236; *a*_P_ = 3.572	2.0055	[59,60]
**^•^DIPPMPO_degr_**	1.469	*a*_H_^β^ = 2.147; *a*_P_ = 4.830	2.0055	–
**^•^POBN–OH**	1.508	*a*_H_^β^ = 0.169	2.0057	[44]
**^•^POBN_degr_**	1.461	*a*_H_^β^ = 1.413	2.0055	[61]
**Water/DMSO (5:1 v:v)**				
**^•^DMPO–OH**	1.469	*a*_H_^β^ = 1.358; *a*_H_^γ^ = 0.067	2.0057	[56]
**^•^DMPO–CH_3_**	1.588	*a*_H_^β^ = 2.250	2.0055	[50]
**^•^DBNBS–CH_3_**	1.434	*a*_H_(3H) = 1.331; *a*_H_(2H*^m^*) = 0.069; $a_{{}^{\mathrm{13}}C}$ = 0.929	2.0063	[50,62]
**^•^DBNBS–CD_3_**	1.434	*a*_D_(3D) = 0.201; *a*_H_(2H*^m^*) = 0.070	2.0063	[50]

Symbols β and γ denote the position of the interacting hydrogen nuclei.

**Figure 5:**
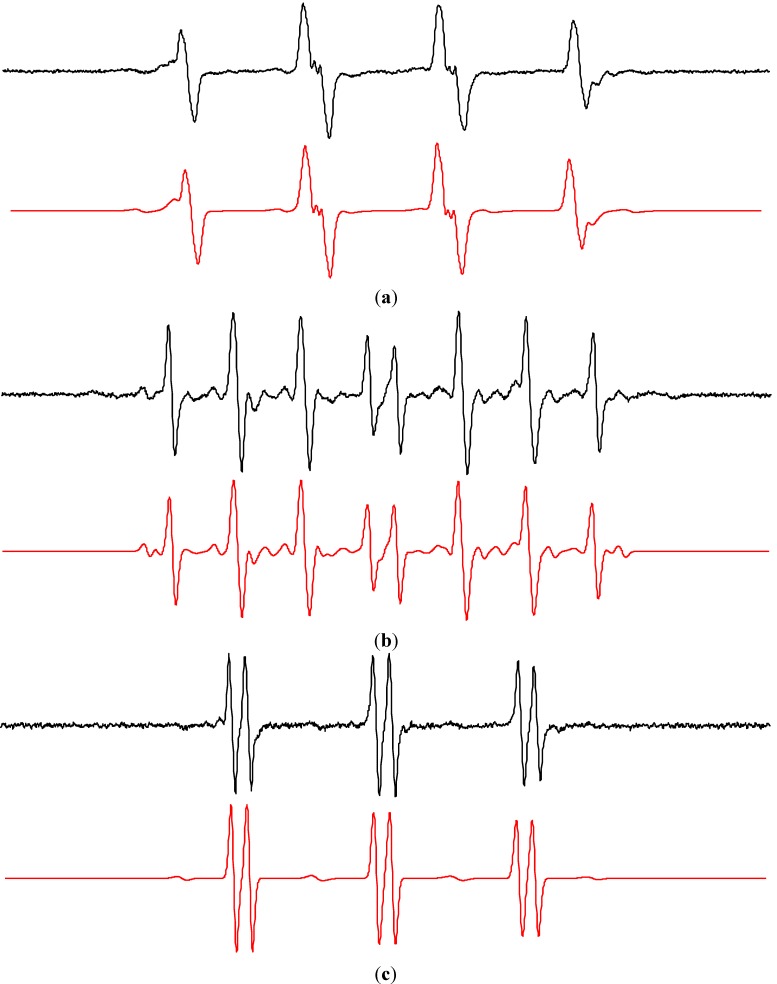
Experimental (**black**) and simulated (**red**) EPR spectra obtained upon irradiation (λ_max_ = 365 nm; irradiance 15 mW∙cm^−2^; exposure 400 s) of the aerated aqueous TiO_2_ P25 suspensions in the presence of various spin trapping agents (TiO_2_ concentration 0.167 mg∙mL^−1^): (**a**) EMPO (*SW* = 8 mT; *c*_0,EMPO_ = 0.05 M); (**b**) DIPPMPO (*SW* = 16 mT; *c*_0,DIPPMPO_ = 0.035 M); (**c**) POBN (*SW* = 8 mT; *c*_0,POBN_ = 0.05 M). Simulations represent linear combinations of corresponding spin-adducts (hfcc parameters listed in Table 2): (a) *trans*-^•^EMPO–OH (relative concentration in %, 68), *cis*-^•^EMPO-OH (28), ^•^EMPO_degr_ (4); (b) *trans*-^•^DIPPMPO-OH (77), *cis*-^•^DIPPMPO-OH (10), ^•^DIPPMPO_degr_ (13); (c) ^•^POBN-OH (92), ^•^POBN_degr_ (8).

The application of 5-(ethoxycarbonyl)-5-methyl-1-pyrroline *N*-oxide (EMPO), 5-(diisopropoxyphosphoryl)-5-methyl-1-pyrroline *N*-oxide (DIPPMPO) and α-(4-pyridyl-1-oxide)-*N*-*tert*-butylnitrone (POBN) spin trapping agents suitable for the detection of oxygen-centered radicals in the aerated aqueous TiO_2_ suspensions upon exposure confirmed the dominant generation of hydroxyl radical spin-adducts (Figure 5). The chiral centre in EMPO and DIPPMPO molecules may result in the production of *trans* and *cis* spin-adduct diastereoisomers. Indeed, the simulation analysis of the corresponding experimental EPR spectra summarized in Table 2, revealed the superposition of two individual EPR signals belonging to hydroxyl radical spin-adduct diastereoisomers. Additionally, low-intensity EPR signals of radical intermediates originating from the spin traps decomposition were detected as the carbon-centered spinadducts (^•^EMPO_degr_, ^•^DIPPMPO_degr_) or the four-line signal of hydroxy *tert*-butylnitroxide (^•^POBN_degr_) [61].

Previously, the photoinduced generation of O_2_^•−^ on the irradiated TiO_2_ nanoparticles was confirmed by low temperature EPR measurements below 160 K [25,31,32,34,52]. Even though the spin trapping agents EMPO and DIPPMPO were specially designed for the detection of superoxide radical anion in aqueous media and biological systems [60,63,64,65,66], the EPR signals reflecting the presence of ^•^EMPO-O_2_^−^/O_2_H and ^•^DIPPMPO-O_2_^−^/O_2_H spin-adducts were not found in the irradiated aqueous TiO_2_ suspensions (Figure 5). Most probably, under the given experimental conditions, the superoxide radical anions are preferably transformed to hydrogen peroxide by a disproportionation with protons [24,26,67]. Moreover, a significantly lower rate constants for the addition of O_2_^•−^/^•^O_2_H to the nitrone spin traps may cause the limited production of spin-adducts [46,48]. The rapid transformation of superoxide radical anions, as well as their very slow reaction with nitrone spin traps caused that at room temperature in aerated aqueous TiO_2_ suspensions superoxide detection using conventional spectroscopic techniques failed, and only chemiluminescence with luminol or luciferin analog was applied as a suitable experimental method [68,69,70].

### 2.2. Spin Trapping in Non-Aqueous TiO_2_ Suspensions

EPR spin trapping investigations of reactive radicals produced in the irradiated TiO_2_ dispersions so far were focused mainly on aqueous systems and on the detection of hydroxyl radicals [20,31,35,36,37,49]. Analogous experiments performed in organic solvents may provide interesting information concerning the radical intermediates generated [13,71,72,73,74], consequently we carried out spin trapping experiments with TiO_2_ nanoparticles dispersed in DMSO, acetonitrile (ACN), methanol and ethanol. The generation of electron-hole pairs upon TiO_2_ irradiation and their consecutive reactions resulting in the free radicals formation are substantially influenced by the solvent properties [54,75,76]. The increased solubility of molecular oxygen plays an important role in these processes (Table 3), together with the stabilization effect of the aprotic solvents on the superoxide radical anions and the reactivity of holes and hydroxyl radicals with the solvents (Table 1).

**Table 3:** Solubility of molecular oxygen in various solvents at 25 °C.

Solvent	$c_{O_{2}}$, mM	Reference
Water	1.0	[77]
Dimethylsulfoxide	2.1	[78]
Acetonitrile	8.1	[78]
Methanol	9.4–10.3	[79]
Ethanol	7.5–11.6	[79]

#### 2.2.1. Dimethylsulfoxide

**Figure 6:**
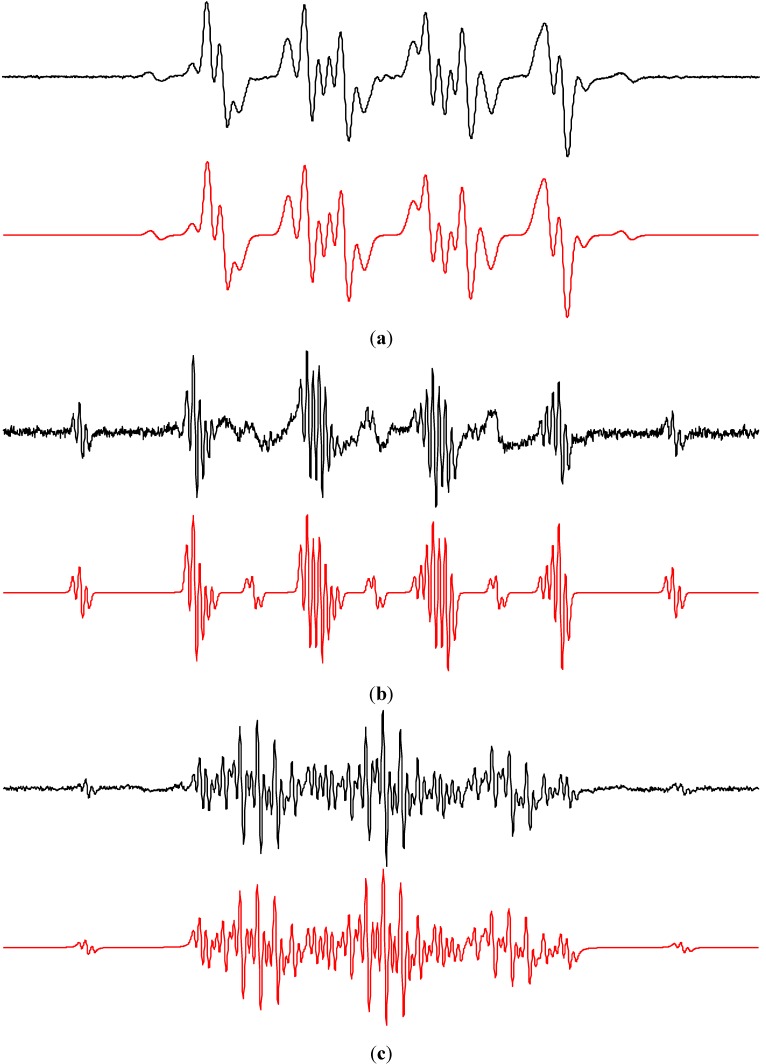
Experimental (**black**) and simulated (**red**) EPR spectra (*SW* = 8 mT) obtained upon irradiation (λ_max_ = 365 nm; irradiance 15 mW∙cm^−2^; exposure 400 s) of the aerated TiO_2_ P25 DMSO suspensions in the presence of various spin trapping agents (TiO_2_ concentration 0.167 mg∙mL^−1^): (**a**) DMPO (*c*_0,DMPO_ = 0.035 M); (**b**) DBNBS (*c*_0,DBNBS_ = 0.008 M); (**c**) DBNBS using DMSO-*d*_6_ (83% vol.). The spin Hamiltonian parameters of corresponding spin-adducts are listed in Table 4. (**a**) ^•^DMPO–O_2_^−^ (relative concentration in %, 46), ^•^DMPO–OCH_3_ (46), ^•^DMPO–OR (6), ^•^DMPO–CH_3_ (2); (**b**) ^•^DBNBS–CH_3_ (90), ^•^DBNBS–SO_3_^−^ (10); (**c**) ^•^DBNBS–CD_3_ (86), ^•^DBNBS–CH_3_ (14).

The EPR spectra monitored upon UVA photoexcitation of TiO_2_ suspensions in aerated DMSO (Figure 6a) differ from those found when water was used as a solvent (Figure 3a), and the dominating signals represent spin-adducts ^•^DMPO-O_2_^−^ and ^•^DMPO-OCH_3_ with the spin Hamiltonian parameters summarized in Table 4. The superoxide radical anion stabilization in the aprotic solvent explains its favourable generation and consequently also trapping [24,25]. The production of ^•^DMPO-OCH_3_ adduct is initiated by the oxidation of hydroxide anions or water molecules adsorbed on the titanium dioxide surface producing reactive hydroxyl radicals, which immediately attack the DMSO solvent forming methyl radicals (Equation (7)), as shown above in the mixed water/DMSO solvent (Figure 4). The rapid reaction of methyl radicals with molecular oxygen results in the generation of peroxomethyl radicals serving as a source of ^•^DMPO–OCH_3_ spin-adducts (Equations (9)–(13)) [73]. Further low-intensity oxygen-centered spin-adduct assigned to ^•^DMPO–OR originates from the solvent and most probably represents ^•^DMPO–OCH_2_S(O)CH_3_:

^•^CH_3_ + O_2_ → CH_3_OO^•^ (9)

DMPO + CH_3_OO^•^ → ^•^DMPO–OOCH_3_ (10)

2 ^•^DMPO–OOCH_3_ → O_2_ + 2 ^•^DMPO–OCH_3_ (11)

2 CH_3_OO^•^ → 2 CH_3_O^•^ + O_2_ (12)

DMPO + CH_3_O^•^ → ^•^DMPO–OCH_3_ (13)

The photoinduced generation of methyl radicals upon the irradiation of titania-DMSO dispersions was confirmed by the nitroso spin trapping agent DBNBS, suitable for the identification of carbon-centered radicals, as typical signal of ^•^DBNBS-CH_3_ and ^•^DBNBS-CD_3_ (using DMSO-*d*_6_) were detected (Figure 6b,c). Depending on the experimental conditions the spin trap decomposition may occur during the photocatalytic processes demonstrated by the generation of ^•^DBNBS-SO_3_^−^ (Table 4).

#### 2.2.2. Acetonitrile

**Figure 7:**
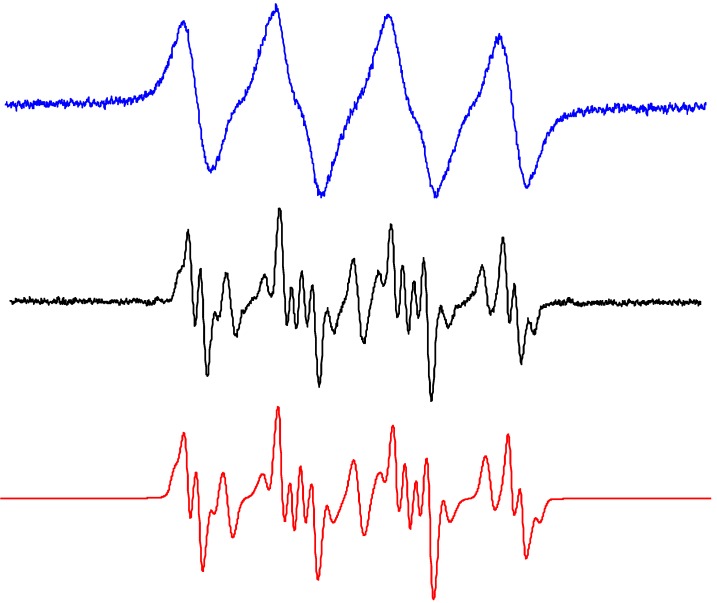
Experimental (**blue**) EPR spectra (*SW* = 8 mT) obtained upon irradiation (λ_max_ = 365 nm; irradiance 15 mW∙cm^−2^; exposure 400 s) of the aerated TiO_2_ P25 (0.167 mg∙mL^−1^) suspensions in ACN containing DMPO spin trap (*c*_0,DMPO_ = 0.035 M), along with EPR spectra measured after post-radiation saturation with argon (**black**) and their simulations (**red**). The spin Hamiltonian parameters of corresponding spin-adducts are listed in Table 4. ^•^DMPO-O_2_^−^ (rel. conc. in %, 60), ^•^DMPO–OH (9), ^•^DMPO-OCH_3_ (23), ^•^DMPO_degr_ (8).

Due to the increased solubility of molecular oxygen in ACN (Table 3) the EPR spectra monitored in the irradiated systems TiO_2_/DMPO/ACN/air represent a four-line EPR signal with significantly broadened spectral lines (Figure 2c, Figure 7 blue line). Consequently, to obtain spectra suitable for the identification of spin-adduct parameters, the saturation of the exposed sample with argon is necessary. The experimental EPR spectrum recorded immediately after a post-radiation Ar-saturation and EPR spectrometer re-tuning, along with its simulation is shown in Figure 7 (black and red lines). Acetonitrile as an aprotic solvent stabilizes superoxide radical anions, consequently the spin-adduct ^•^DMPO-O_2_^−^ dominates the EPR spectrum. The use of dried ACN solvent indicates that the hydroxyl radicals are generated by the oxidation of OH^−^/H_2_O adsorbed on the TiO_2_ surface via the photogenerated holes [80]. A lower reactivity of the photogenerated hydroxyl radicals towards acetonitrile (Table 1) allows the hydroxyl radicals to be trapped by DMPO and the ^•^DMPO-OH was found in the spectra (Figure 7). The formation of ^•^DMPO-OCH_3_ most probably relates to the interaction of hydroxyl radicals with the solvent [81] producing CH_3_OO^•^ radicals trapped as the ^•^DMPO-OCH_3_ spin-adducts [73]. The spin Hamiltonian parameters of the individual spin-adducts elucidated by the simulation of experimental EPR spectra obtained in TiO_2_/DMPO/ACN/air are summarized in Table 4.

Additional experiments with nitrosodurene (ND) spin trap were performed in order to identify the structure of the carbon-centered radicals produced during the exposure of TiO_2_/ACN/air. The EPR spectra measured upon continuous irradiation (spectra not shown) revealed the presence of a nine-line signal of ^•^ND–CH_2_CN, produced via the interaction of ^•^OH with ACN [54], and a broad, three-line signal of ND^•+^ generated by the spin trap oxidation (Table 4).

#### 2.2.3. Methanol and Ethanol

The protic solvents methanol and ethanol are characterized with increased concentration of dissolved molecular oxygen (Table 3), and these solvents are well-known as efficient scavengers of photogenerated holes [82]. By their application radical intermediates created via interaction with both photogenerated charge carriers may be observed, *i.e*., electrons are scavenged by molecular oxygen forming O_2_^•−^ and holes react with alcohols producing the primary alkoxy radical species, ^•^OCH_3_ and ^•^OCH_2_CH_3_ (Equations (14) and (15)) [12,13,62].

CH_3_OH + h^+^ → CH_3_O^•^ + H^+^ (14)

CH_3_CH_2_OH + h^+^ → CH_3_CH_2_O^•^ + H^+^ (15)

**Table 4:** Spin Hamiltonian parameters (hyperfine coupling constants and *g*-values) of spin-adducts elucidated from the simulations of experimental EPR spectra obtained upon UVA irradiation (λ_max_ = 365 nm) of aerated TiO_2_ P25 suspensions in organic solvents in the presence of spin traps.

Spin-Adduct	Hyperfine Coupling Constants (mT)		*g*-Value	Reference
	*a*_NO_	*a*_i_		
**DMSO**				
**^•^DMPO–O_2_^−^**	1.287	*a*_H_^β^ = 1.041; *a*_H_^γ^ = 0.139	2.0057	[25,50,83]
**^•^DMPO–OCH_3_**	1.329	*a*_H_^β^ = 0.808; *a*_H_^γ^ = 0.164	2.0057	[50,84]
**^•^DMPO–OR**	1.301	*a*_H_^β^ = 1.464	2.0057	[84]
**^•^DMPO–CH_3_**	1.462	*a*_H_^β^ = 2.093	2.0056	[50]
**^•^DBNBS–CH_3_**	1.337	*a*_H_(3H) = 1.211; *a*_H_(2H*^m^*) = 0.067	2.0064	[50]
**^•^DBNBS–CD_3_**	1.334	*a*_D_(3D) = 0.183; *a*_H_(2H*^m^*) = 0.067	2.0064	[50]
**^•^DBNBS–SO_3_^−^**	1.295	*a*_H_(2H*^m^*) = 0.054	2.0064	[85]
**ACN**				
**^•^DMPO–O_2_^− #^**	1.296	*a*_H_^β^ = 1.044; *a*_H_^γ^ = 0.133	2.0057	[50,74]
**^•^DMPO–OH ^#^**	1.382	*a*_H_^β^ = 1.200; *a*_H_^γ^ = 0.080	2.0057	[74]
**^•^DMPO–OCH_3_^#^**	1.312	*a*_H_^β^ = 0.796; *a*_H_^γ^ = 0.179	2.0057	[74]
**^•^DMPO_degr_^#^**	1.479		2.0056	–
**^•^ND–CH_2_CN**	1.342	*a*_H_(2H) = 0.977	2.0057	[86,87]
**ND^•+^**	2.608		2.0057	[50]
**Methanol**				
**^•^DMPO–O_2_^− #^**	1.376	*a*_H_^β^ = 0.963; *a*_H_^γ^ = 0.132	2.0057	[50]
**^•^DMPO–OCH_3_^#^**	1.363	*a*_H_^β^ = 0.775; *a*_H_^γ^ = 0.167	2.0057	[50]
**^•^DMPO–OCH_2_OH ^#^**	1.414	*a*_H_^β^ = 1.266; *a*_H_^γ^ = 0.075	2.0057	[50]
**^•^DMPO–CH_2_OH ^#^**	1.506	*a*_H_^β^ = 2.116	2.0056	[50]
**^•^DMPO_degr_^#^**	1.523		2.0056	–
**^•^ND–CH_2_OH**	1.387	*a*_H_(2H) = 0.771	2.0057	[62,86,87]
**Ethanol**				
**^•^DMPO–O_2_^− #^**	1.322	*a*_H_^β^ = 1.050; *a*_H_^γ^ = 0.133	2.0057	[50]
**^•^DMPO–OCH_2_CH_3_^#^**	1.356	*a*_H_^β^ = 0.761; *a*_H_^γ^ = 0.174	2.0057	[50]
**^•^DMPO–OR ^#^**	1.470	*a*_H_^β^ = 1.094; *a*_H_^γ^ = 0.090	2.0057	[50]
**^•^DMPO–CR_1_^#^**	1.481	*a*_H_^β^ = 2.195	2.0056	[50]
**^•^DMPO–CR_2_^#^**	1.534	*a*_H_^β^ = 2.215	2.0056	[50]
**^•^ND–CH(CH_3_)OH**	1.398	*a*_H_ = 0.702	2.0057	[62,86,87]
**^•^ND–CH_3_**	1.452	*a*_H_(3H) = 1.345	2.0057	[62,86,87]

^#^: post-radiation saturation with argon. Symbols β and γ denote the position of the interacting hydrogen nuclei.

**Figure 8:**
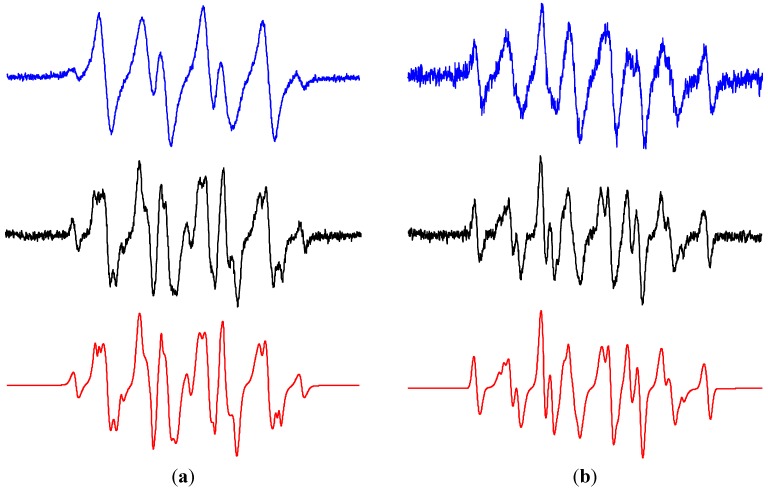
Experimental (**blue**) EPR spectra (*SW* = 8 mT) obtained upon irradiation (λ_max_ = 365 nm; irradiance 15 mW∙cm^−2^; exposure 400 s) of the aerated TiO_2_ P25 (0.167 mg∙mL^−1^) suspensions in organic solvents containing DMPO spin trap (*c*_0,DMPO_ = 0.035 M), along with EPR spectra measured after post-radiation saturation with argon (**black**) and their simulations (**red**): (**a**) methanol; (**b**) ethanol. The spin Hamiltonian parameters of corresponding spin-adducts are listed in Table 4. (a) ^•^DMPO–O_2_^−^ (rel. conc. in %, 33),^•^DMPO–OCH_3_ (35), ^•^DMPO–OCH_2_OH (21), ^•^DMPO–CH_2_OH (7), ^•^DMPO_degr_ (4); (b) ^•^DMPO–O_2_^−^ (17), ^•^DMPO–OCH_2_CH_3_ (59), ^•^DMPO–OR (10), ^•^DMPO–CR_1_ (9), ^•^DMPO–CR_2_ (5).

These oxygen-centered radical species can be easily identified using the DMPO spin trap. Due to the higher concentration of dissolved oxygen in these solvents, the EPR spectra of spin-adducts measured in aerated methanol and ethanol TiO_2_ suspensions are characterized by a significant line broadening, which hinders a detailed simulation analysis (Figure 8a,b blue lines). However, the post-radiation saturation of the TiO_2_ suspensions with argon, and subsequent measurement of EPR spectra provide the EPR signals of sufficient quality (Figure 8a,b black lines).

Simulations of the EPR spectra measured using this experimental procedure in the system TiO_2_/DMPO/methanol revealed the presence of individual DMPO spin-adducts corresponding to ^•^DMPO-O_2_^−^, ^•^DMPO-OCH_3_, ^•^DMPO-CH_2_OH, ^•^DMPO-OCH_2_OH and a triplet signal assigned to the DMPO degradation product (Figure 8a red line). The spin Hamiltonian parameters of the identified spin-adducts are gathered in Table 4. We assume that the radical species ^•^CH_2_OH and ^•^OCH_2_OH are produced via complex reactions of ^•^OCH_3_ radicals with methanol molecules and molecular oxygen [62], an alternative mechanism of ^•^CH_2_OH generation represents the hydrogen abstraction from methanol via hydroxyl radicals (Table 1, Equations (16) and (17)).

CH_3_O^•^ + CH_3_OH → CH_3_OH + ^•^CH_2_OH (16)

CH_3_OH + ^•^OH → ^•^CH_2_OH + H_2_O (17)

Simulation of the EPR spectra obtained in the irradiated suspensions TiO_2_/DMPO/ethanol after the saturation with argon evidenced the presence of spin-adducts characteristic for ^•^DMPO-O_2_^−^, ^•^DMPO-OCH_2_CH_3_, ^•^DMPO-OR, and two carbon-centered spin-adducts with slightly differing hyperfine coupling constants (Figure 8b, Table 4). Nitrosodurene spin trapping agent was used in the analogous experiments to identify the carbon-centered radicals in the irradiated methanol or ethanol TiO_2_ suspensions (spectra not shown). In methanol only the generation of ^•^ND-CH_2_OH was evidenced. The EPR spectra monitored in TiO_2_/ethanol suspensions are compatible with ^•^ND-CH(OH)CH_3_ and ^•^ND-CH_3_ spin-adducts in good accordance with the reactions of ethoxy, 1-hydroxyethyl and 2-hydroxyethyl radicals in ethanol (Equations (18)–(20)) published previously [62], and also with two DMPO carbon-centered spin-adducts detected:

CH_3_CH_2_O^•^ + CH_3_CH_2_OH → CH_3_CH_2_OH + CH_3_^•^CHOH (18)

CH_3_^•^CHOH + CH_3_CH_2_OH → ^•^CH_2_CH_2_OH + CH_3_CH_2_OH (19)

CH_2_^•^CH_2_OH → ^•^CH_3_ + CH_2_O (20)

### 2.3. Oxidation of Sterically Hindered Amine in TiO_2_ Suspensions

The irradiation of titanium dioxide nanoparticles in the presence of molecular oxygen results in the generation of singlet oxygen, but the specific mechanism of ^1^O_2_ formation is not straightforward and alternative reaction pathways have been suggested [88,89,90]. The direct detection of ^1^O_2_ is based on the phosphorescence measurement at 1270 nm corresponding to the radiative transition O_2_(^1^∆_g_)→O_2_(^3^Σ_g_) [89,91]. The principle of the indirect techniques of ^1^O_2_ monitoring is the specific reaction with an organic compound generating a product detectable by a suitable method [92], supported by the application of ^1^O_2_ scavengers and traps, or using the effect of deuterated solvents. The photoinduced formation of singlet oxygen in the homogeneous systems is frequently monitored also by EPR spectroscopy, detecting the generation of nitroxide radicals derived from 4(R)-2,2,6,6-tetramethylpiperide *N*-oxyl (R = hydroxy, oxo) produced by the oxidation of corresponding sterically hindered amines (SHA) [84,93,94]. Although this method is widely used for the singlet oxygen detection, many questions arise concerning its selectivity [95]. Particular problems may appear when a numerous ROS or other reactive species are formed in the studied system and their interaction with SHA cannot be excluded, e.g., in the irradiated TiO_2_ suspensions. The detailed analysis of paramagnetic species generated in homogeneous ACN solutions and TiO_2_ suspensions in the presence SHA and ROS was performed previously in our laboratory [74].

**Figure 9:**
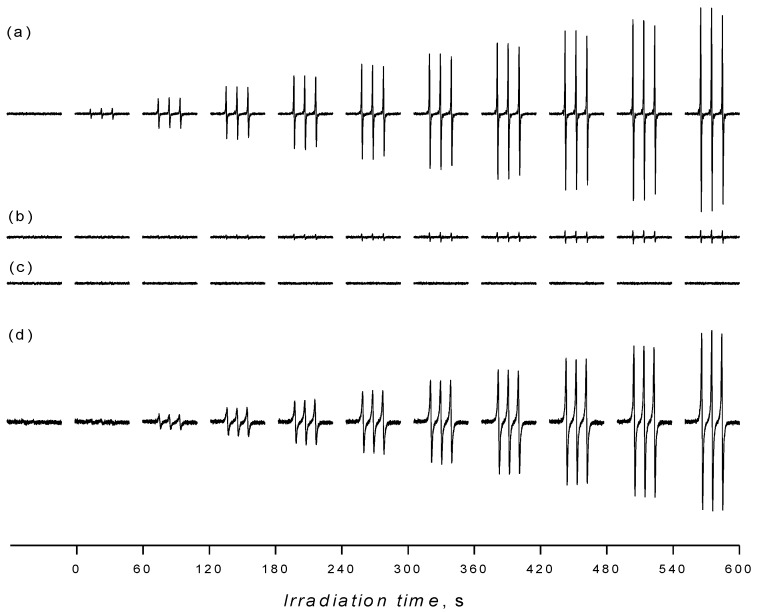
The sets of individual EPR spectra (*SW* = 8 mT) monitored upon continuous UVA irradiation (λ_max_ = 365 nm; irradiance 15 mW∙cm^−2^) of aerated TiO_2_ P25 suspensions in the presence of sterically hindered amine TMPO: (**a**) oxygenated water; (**b**) oxygenated water saturated with β-carotene; (**c**) oxygenated mixed solvent water/DMSO (5:1 v:v); (**d**) aerated ACN. TiO_2_ concentration 0.167 mg∙mL^−1^, *c*_0,TMPO_ = 0.008 M.

The concentration of molecular oxygen in aqueous TiO_2_ suspensions play an important role during the oxidation of 4-oxo-2,2,6,6-tetramethylpiperidine (TMPO) to the radical product 2,2,6,6-tetramethylpiperidine *N*-oxyl (Tempone; *a*_N_ = 1.617 mT, *a*_13C_(4^13^C) = 0.610 mT; *g* = 2.0054). In the photoexcited system TiO_2_/TMPO/water/air the concentrations of Tempone was very low, and the prolonged irradiation led to a total disappearance of the EPR signal (data not shown). However, the saturation of the aqueous TiO_2_ suspension by oxygen led to the continuous growth of the EPR signal of Tempone (*a*_N_ = 1.479 mT; *g* = 2.0057) as shown in Figure 9a. The addition of sodium azide, a widely used water-soluble singlet oxygen quencher, to the TiO_2_/TMPO/water/O_2_ systems completely suppressed the Tempone generation. However this result should be very carefully analyzed, since besides the singlet oxygen, azide anions also react very fast with the hydroxyl radicals (*k* = 1.4 × 10^10^ M^−1^ s^−1^ [55]) producing the azide radical ^•^N_3_ [96] detected here as the corresponding spin-adduct ^•^DMPO-N_3_ in the photoexcited system TiO_2_/DMPO/water/NaN_3_/air (Table 2). Despite the limited water solubility of β-carotene, an analogous inhibition of TMPO photooxidation was observed also when β-carotene as an effective singlet oxygen quencher [97] was added to the TiO_2_ suspensions (Figure 9b). However, due to the lack of specificity the alternative reaction pathways of β-carotene with the radical species generated in the irradiated titania suspensions must be considered [98]. The role of hydroxyl radicals in the SHA oxidation was further demonstrated in the mixed solvent containing DMSO, where the total inhibition of Tempone formation was found, due to the effective scavenging of hydroxyl radicals by DMSO (Figure 9c). The increased concentration of dissolved molecular oxygen in acetonitrile (Table 3), as well as longer lifetime of ^1^O_2_ in this solvent (Table 1) resulted in the effective oxidation of TMPO to Tempone. The higher ^3^O_2_ concentration in the systems TiO_2_/TMPO/ACN/air is reflected also in the spectral linewidth growth with not-resolved ^13^C-satellites (Figure 9d).

## 3. Experimental Section

The commercial titanium dioxide Aeroxide^®^ P25 (Evonic Degussa, Essen, Germany) was used and stock suspensions containing 1 mg TiO_2_ mL^−1^ were prepared in redistilled water, dimethylsulfoxide (Merck, Darmstadt, Germany, SeccoSolv^®^, max. 0.025% H_2_O), acetonitrile (Merck, SeccoSolv^®^, max. 0.005% H_2_O), methanol (spectroscopic grade, Lachema, Brno, Czech Republic), and ethanol (for UV spectroscopy, MikroChem, Pezinok, Slovak Republic). The isotopically enriched water-^17^O (20%–24.9% atom. ^17^O) and deuterated DMSO-*d*_6_, both from Sigma-Aldrich (Buchs, Switzerland), were used as co-solvents. The stock TiO_2_ suspensions were homogenized for 1 min using ultrasound (Ultrasonic Compact Cleaner TESON 1; Tesla, Piešťany, Slovak Republic). The spin trapping agent 5,5-dimethyl-1-pyrroline *N*-oxide (DMPO, Sigma-Aldrich) was distilled prior to use. 5-(Diisopropoxyphosphoryl)-5-methyl-1-pyrroline *N*-oxide (DIPPMPO, Enzo Life Sciences, Farmingdale, NY, USA), 5-(ethoxycarbonyl)-5-methyl-1-pyrroline *N*-oxide (EMPO; Enzo Life Sciences), α-(4-pyridyl-1-oxide)-*N*-*tert*-butylnitrone (POBN; Janssen Chimica, Geel, Belgium), 2,3,5,6,-tetramethylnitrosobenzene (nitrosodurene, ND, Sigma-Aldrich) and 3,5-dibromo-4-nitrosobenzene sulfonate (DBNBS, Sigma-Aldrich) were used without extra purification. All spin traps were stored at −18 °C. The stock solutions of the spin trapping agents were prepared in studied solvents, apart from the ND, characteristic with a limited solubility in polar solvents, which was applied in a saturated suspension directly before the specific experiments. The concentrations of spin traps applied were chosen in order to minimize the undesired photochemical reactions of the spin traps and to gain the effective trapping of photogenerated radical species. The sterically hindered amine 4-oxo-2,2,6,6-tetramethylpiperidine (TMPO, Merck-Schuchardt, Hohenbrunn, Germany) was used as supplied. Sodium azide (analytical grade, Sigma-Aldrich) and β-carotene (UV grade, Sigma-Aldrich) were applied as the singlet oxygen quenchers. Concentrations of the photogenerated paramagnetic species were determined using solutions of 4-oxo-2,2,6,6-tetramethylpiperidine *N*-oxyl (Tempone, Sigma-Aldrich) as the calibration standards.

The TiO_2_ P25 suspensions containing the spin trapping agent or the TMPO was mixed and carefully saturated with air or oxygen using a slight gas stream immediately before the EPR measurement. So prepared samples were transferred to a small quartz flat cell (WG 808-Q, optical cell length 0.04 cm; Wilmad-LabGlass, Vineland, NJ, USA) optimized for the TE_102_ cavity (Bruker, Rheinstetten, Germany) of the spectrometer X-band EPR spectrometer (EMXplus, Bruker). During the EPR photochemical experiments the samples were irradiated at 295 K directly in the EPR resonator, and the EPR spectra were recorded *in situ* during a continuous photoexcitation or after a defined exposure. As an irradiation source a UV LED monochromatic radiator (λ_max_ = 365 nm; Bluepoint LED, Hönle UV Technology, Gräfelfing/München, Germany) was used. The irradiance value (λ_max_ = 365 nm; 15 mW∙cm^−2^) within the EPR cavity was determined using a UVX radiometer (UVP, Upland, CA, USA). In some cases, argon saturation needed to be applied after the irradiation of the aerated suspensions prior to the subsequent EPR experiment to get better resolved spectra by suppressing the line-broadening effect of molecular oxygen.

Typical EPR spectrometer settings in a standard photochemical experiment were: microwave frequency, ~9.424 GHz; microwave power, 10.53 mW; center field, 335.6 mT; sweep width, 8–16 mT; gain, 1 × 10^5^ to 1 × 10^6^; modulation amplitude, 0.05–0.1 mT; scan, 20 s; time constant, 10.24 ms. The *g*-values (±0.0001) were determined using a built-in magnetometer. The EPR spectra so obtained were analyzed and simulated using the Bruker software WinEPR and SimFonia and the Winsim2002 [99].

## 4. Conclusions

The EPR spin trapping experiments using a variety of spin trapping agents (DMPO, EMPO, DIPPMPO, POBN, DBNBS and ND) were performed to identify reactive intermediates formed upon irradiation of TiO_2_ suspended in water and organic solvents. The role of water in the photoinduced generation of the hydroxyl radical spin-adduct ^•^DMPO-OH in aerated aqueous TiO_2_ systems was evidenced using ^17^O-enriched water. Application of a water-soluble nitroso spin trapping agent DBNBS confirmed the production of methyl radicals when DMSO was added to the aqueous TiO_2_ suspensions and the addition of DMSO-*d*_6_ revealed also the origin of these radicals. The photoexcitation of TiO_2_ in non-aqueous solvents (DMSO, ACN, methanol and ethanol) in the presence of spin trapping agents showed the stabilization of superoxide radical anions generated via electron transfer reaction to molecular oxygen, as well as the production of various oxygen- and carbon-centered radicals from the solvents. The oxidation of sterically hindered amine TMPO to radical Tempone via ROS was monitored in aqueous and acetonitrile TiO_2_ suspensions.

The results obtained demonstrate that indirect EPR spectroscopy techniques represent valuable tools for the characterization of radical intermediates generated in irradiated TiO_2_ suspensions. However, a careful selection of the experimental conditions and a precise analysis of the experimental EPR spectra considering alternative reaction pathways is an important aspect of any successful application of these indirect techniques in the characterization of TiO_2_ photoactivity.

## Supplementary Materials

Supplementary materials can be accessed at: http://www.mdpi.com/1420-3049/19/11/17279/s1.

## Supplementary Files

Supplementary File 1
