# Supplementary material for: Radical Intermediates in Photoinduced Reactions on TiO2 (An EPR Spin Trapping Study)
Source: Molecules. 2014 Oct 28;19(11):17279–304. doi: 10.3390/molecules191117279 (PMC6271711; doi:10.3390/molecules191117279)

Supplementary Materials

Figure S1. Experimental EPR spectrum (*SW* = 8 mT) obtained after argon saturation of methanol TiO_2 P25 suspension (0.167 mg·mL^{-1}) irradiated under air in the presence of DMPO spin trap ($c_{0,DMPO}$ = 0.035 M), along with the simulated spectrum constructed from the EPR signals of the individual spin-adducts. The spin Hamiltonian parameters of spin-adducts are summarized in Table 4.

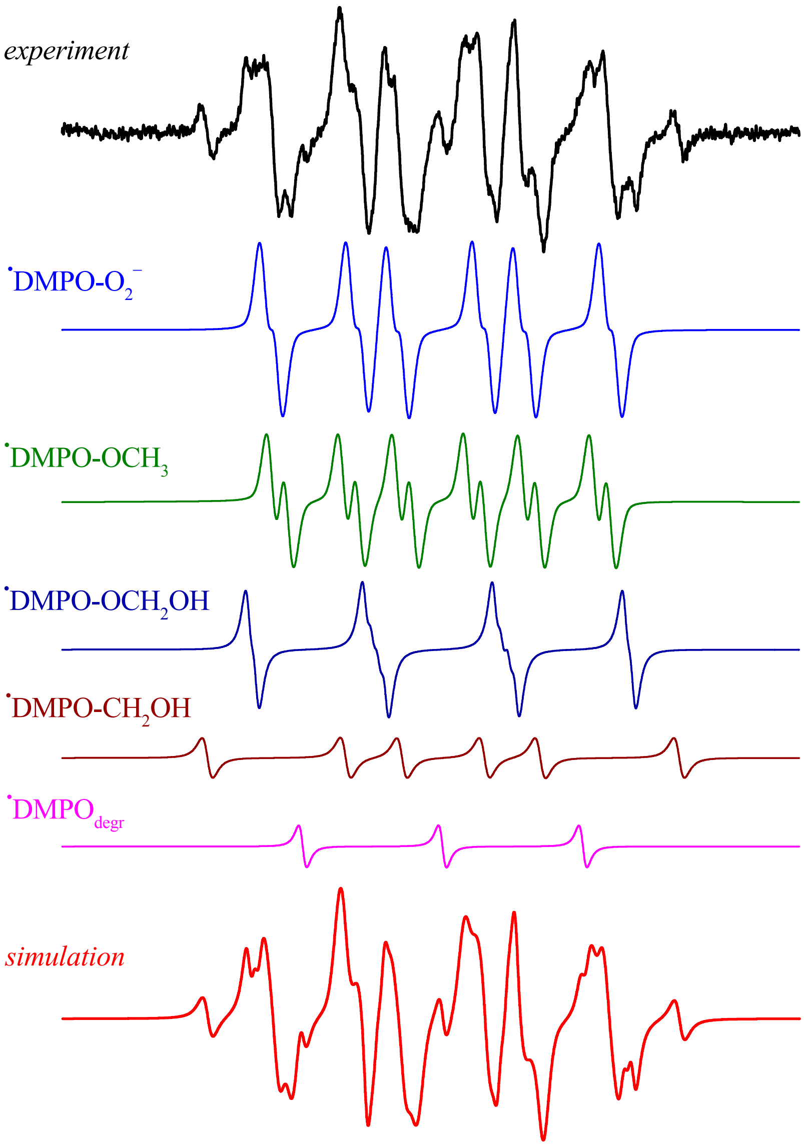

Figure S2. Experimental EPR spectrum (SW = 8 mT) obtained after argon saturation of ethanol TiO_2 P25 suspension (0.167 mg·mL^{-1}) irradiated under air in the presence of DMPO spin trap ($c_{0,DMPO}$ = 0.035 M), along with the simulated spectrum constructed from the EPR signals of the individual spin-adducts. The spin Hamiltonian parameters of spin-adducts are summarized in Table 4.

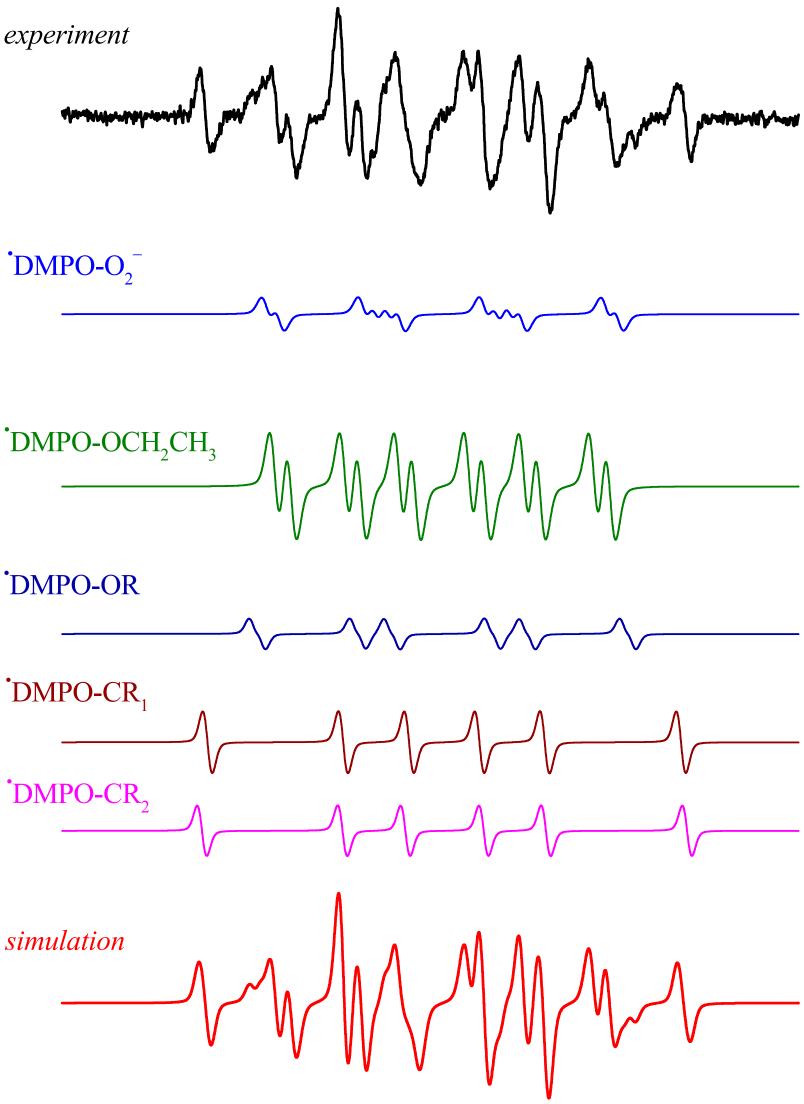

Supplement: Supplementary File 1 [file molecules-19-17279-s001.pdf]
